# The Prognostic Model and Drug Sensitivity of *LKB1*-Mutant Lung Adenocarcinoma Based on Immune Landscape

**DOI:** 10.3389/fmolb.2022.756772

**Published:** 2022-06-02

**Authors:** Guanghui Wang, Haotian Zheng, Xiaogang Zhao, Yadong Wang, Yukai Zeng, Jiajun Du

**Affiliations:** ^1^ Institute of Oncology, Shandong Provincial Hospital, Shandong University, Jinan, China; ^2^ Department of Thoracic Surgery, Shandong Provincial Hospital, Shandong University, Jinan, China; ^3^ Department of Thoracic Surgery, The Second Hospital of Shandong University, Jinan, China

**Keywords:** LKB1, lung adenocarcinoma, prognostic model, TCGA, Immune, drug sensitivity

## Abstract

**Background:** Lung cancer is the most common cause of cancer-related deaths worldwide. *LKB1*-mutant lung adenocarcinoma (LUAD) is a unique subtype of this deadly cancer. *LKB1* mutations cause functional changes in a variety of cell processes, including immune functions, that affect prognosis. To date, the potential role of immunity in the prognosis of *LKB1*-mutant LUAD is not well understood.

**Methods:** We systematically analyzed immune-related genes in LUAD samples from The Cancer Genome Atlas (TCGA) database. ESTIMATE and CIBERSORT algorithms were used to explore the immune microenvironment. A prognostic risk model was constructed, and prognostic, immune function, drug sensitivity, and model specificity analyses were performed to identify the effectiveness of the model.

**Results:** Our results showed that *LKB1* mutations suppressed immune function in LUAD. A three-gene signature was constructed to stratify patients into two risk groups. The risk score was an independent predictor for overall survival (OS) in multivariate Cox regression analyses [hazard ratio (HR) > 1, *p* = 0.002]. Receiver operating characteristic (ROC) curve analyses confirmed that the risk score has better performance than clinicopathological characteristics. Functional analysis revealed that the immune status was different between the risk groups. ZM.447439 was an appropriate treatment for the high-risk group of patients. This risk model is only suitable for *LKB1-*mutant tumors; it performed poorly in LUAD patients with wild-type *LKB1*.

**Conclusion:** Our findings indicate the potential role of immunity in *LKB1*-mutant LUAD, providing novel insights into prognosis and guiding effective immunotherapy.

## Introduction

Lung cancer is the most common cause of cancer-related deaths worldwide, with more than 40% of cases being lung adenocarcinoma (LUAD) (Jordan et al., 2017). Genomic alterations have important impacts on tumor cell-intrinsic and non-cell-autonomous cancer hallmarks; accordingly, genomic alterations underlie the molecular and clinical heterogeneity of LUAD (Skoulidis and Heymach, 2019). The gene encoding liver kinase B1 (*LKB1*) is the third most commonly mutated gene in LUAD (after *KRAS* and *TP53*), with approximately 19% of LUAD cases involving *LKB1* mutations (Ding et al., 2008; Cancer Genome Atlas Research, 2014). Moreover, LKB1 is involved in many cell processes essential for cell survival, such as metabolic balance, maintenance of DNA integrity, proliferation, and polarity establishment (Shackelford and Shaw, 2009). Notably, therapeutic approaches targeting oncogene driver mutations, for example, activating mutations in the epidermal growth factor receptor or rearrangements in anaplastic lymphoma kinase, have been recently shown to elicit dramatic clinical responses (Paez et al., 2004; Soda et al., 2007; Bergethon et al., 2012). However, there are no routinely used clinical drugs that specifically target LUAD with *LKB1*-inactivating mutations.

The tumor microenvironment (TME) refers to the local biological environment including cancer cells, stromal cells, and distant recruited cells, such as infiltrating immune cells (myeloid cells and lymphocytes), bone marrow-derived cells, and secreted factors such as cytokines and chemokines (Wang et al., 2017; Zhu et al., 2020). The status of the TME plays a bidirectional role in tumor progression and often impacts the effectiveness of targeted drugs (Topalian et al., 2012; Tamborero et al., 2018). While the complexity of TME components has proven to be a barrier to research, some new algorithms, including ESTIMATE and CIBERSORT, that are based on bulk RNA sequencing have dealt with this problem effectively (Yoshihara et al., 2013; Newman et al., 2015a). These algorithms can quantify the degree of stromal cell and immune cell infiltration in tumor tissues, and their accuracy has been verified in breast cancer and liver cancer models (Newman et al., 2015a).

Recent work has shown that oncogenic mutations shape the TME and determine its immune context (Akbay et al., 2013). Because oncogenes directly activate immune checkpoints to impact immune-evading mechanisms and cause immune suppression (Akbay et al., 2013), immunotherapies for immune checkpoint blockade and other aspects efficiently target tumors and show an association with the survival of cancer patients (Taube et al., 2014; Tumeh et al., 2014; Drerup et al., 2020). It is unclear whether the inactivation of tumor suppressor genes such as *LKB1* exerts similar effects, even though recent studies have opened new perspectives in this field.

Thus, there is an urgent need to elucidate the underlying mechanisms influencing immune activity and the TME in *LKB1*-mutant LUAD and to explore potential prognostic biomarkers. Understanding these factors could provide better therapeutic strategies for LUAD. Furthermore, in addition to conventional biomarkers, the multi-faceted roles of long non-coding RNA (lncRNA) and microRNA (miRNA) in the progression of LUAD, including in determining immune and microenvironmental conditions (Shen et al., 2020; Sun et al., 2020), mean that these molecules may also serve as useful indicators of disease progression. Therefore, we have included all kinds of RNA in our research.

To this end, we aim to explore the impact of *LKB*1 mutations on the immune microenvironment of LUAD and the mechanisms that underlie this impact. In addition, we constructed a prognostic model based on immune-related RNAs to promote the development of therapies in *LKB1*-mutant LUAD and to provide novel prognostic biomarkers.

## Materials and Methods

### Data Downloading and Preprocessing

LUAD gene expression data and clinical information were downloaded from The Cancer Genome Atlas (TCGA) database (https://portal.gdc.cancer.gov), and 14,086 lncRNAs, 19,605 mRNAs, and 2,191 miRNAs were obtained by data annotation. According to the mutation status of *LKB1* obtained from the cBioPortal database (https://www.cbioportal.org), we separated TCGA tumor samples into *LKB1-*mutant (LKB1-mu) and *LKB1-*wild-type (LKB1-wt) groups. A total of 1,959 immune-related genes were retrieved from the ImmPort database (https://www.immport.org/resources), and two immune-related gene sets (immune system process M13664 and immune response M19817) were extracted from the Molecular Signatures Database v4.0 (http://www.broadinstitute.org/gsea/msigdb/index.jsp).

We identified 89 patients with *LKB1* mutations by searching the 31 pan-cancer studies (excluding LUAD) in the cBioPortal database. The miRNA expression data of these patients were obtained from the TCGA database, and 74 non-LUAD *LKB1*-mutated patients were ultimately included as a validation cohort.

### Effect of the *LKB1*-Mutation on the Immune Landscape of Lung Adenocarcinoma

The *LKB1* gene mutation status of LUAD was analyzed by probing the cBioPortal database. The difference in the expression level of *LKB1* between the LKB1-wt and LKB1-mu groups was verified by *t*-test. The Gene Set Enrichment Analysis (GSEA) was performed to explore the biological function regulated by the *LKB1* mutant in LUAD. The stromal score, immune score, and ESTIMATE score were also analyzed by the ESTIMATE algorithm based on transcriptome expression profiles of LUAD to examine the effect of the *LKB1* mutant (Yoshihara et al., 2013). The CIBERSORT algorithm was used to accurately determine the composition of 22 kinds of immune cells in a large tumor sample dataset, and the impact of the *LKB1* mutant on these immune cells was determined (Newman et al., 2015b). The TIMER database (https://cistrome.shinyapps.io/timer/) was used to explore the correlation between *LKB1* and immunity. The differential expression of 46 immune checkpoint genes between LKB1-mu and LKB1-wt cells was further explored.

### Identifying Candidate Genes to Construct a Prognostic Risk Model

Expression levels of mRNAs, lncRNAs, and miRNAs in the LKB1-wt and LKB1-mu groups were analyzed by the edger package with the difference screening parameters |logFC| > 1 and false discovery rate (FDR) < 0.05. Utilizing a total of 1959 immune-related genes extracted from the databases, immune-related lncRNAs were identified according to the criteria of |correlation coefficient| > 0.8 and *p* < 0.001 by the Pearson correlation analysis (Schober et al., 2018). The Kaplan–Meier analysis and univariate Cox regression analysis were used to further identify differentially expressed RNA (DERNA) molecules with prognostic values in *LKB1-*mutant LUAD.

To reduce the risk of overfitting, the LASSO-Cox regression analysis was applied to construct a prognostic model (Tibshirani, 1997; Simon et al., 2011). The LASSO algorithm was used for variable selection, and the penalty parameter (*λ*) for the model was determined by a ten-fold cross validation following the minimum criteria. A multivariate regression analysis was then conducted on *LKB1-*mutant LUAD survival-related genes to obtain a risk gene signature, and the risk score based on the signature was formulated as follows: risk score = expX1 × coefX1 + expX2 × coefX2 +… + expXi × coefXi, where, coefXi represents the synergetic coefficient and expXi represents the relative expression of RNA. The median risk score of all samples was used as the critical value to form high-risk and low-risk groups.

### Survival and Prognostic Analysis of the Prognostic Risk Model

The survival difference between the high-risk and low-risk groups was estimated by the K–M survival analysis. Time-dependent receiver operating characteristic curves were drawn, and the area under the curve (AUC) was calculated to estimate and compare the predictive accuracy for survival time by the risk score and different clinical-pathological factors. Univariate and multivariate Cox regression analyses were performed to find independent prognostic factors. The relationship between the clinicopathological characteristics and risk score was identified by a correlation analysis. A nomogram was then drawn to predict 1-, 3-, and 5-year mortality. This nomogram utilized both clinical information and risk scores because both factors have probable impacts on survival. The predictive performance of the nomogram was identified using calibration curves, K–M survival analyses, and ROC curves.

Because of the inaccessibility of other data relating to *LKB1*-mutated LUAD and the similarity of tumor characteristics in the same mutational background, we used patients with other cancers who had *LKB1* mutations as a validation cohort. K–M survival analyses and ROC curves were used to validate the accuracy of the model.

### Immune Function Analysis of the Prognostic Risk Model

The Pearson correlation analysis was performed to assess the relationship between the risk score and immune cell infiltration. For detailed information about immune functions, the single-sample Gene Set Enrichment Analysis (ssGSEA) was performed to calculate the infiltrating score of 16 immune cells and the activity of 13 immune-related pathways (Rooney et al., 2015). A Principal component analysis (PCA) and t-distributed stochastic neighbor embedding (t-SNE) were performed to explore the model’s ability to distinguish *LKB1-*mutant LUAD patients, based on the expression of genes.

### Drug Sensitivity Analysis of the Prognostic Risk Model

To assess the relationship between the chemotherapeutic response and the risk score, we performed a drug sensitivity analysis by using the pRRophetic package based on gene expression data (Geeleher et al., 2014a; Geeleher et al., 2014b). The pRRophetic algorithm has been used extensively in medical studies (Liu et al., 2021a; Liu et al., 2021b; Liu et al., 2022a; Liu et al., 2022b; Liu et al., 2022c). We further explored the expression of targets of immune checkpoint inhibitors and other drugs between high-risk and low-risk groups to search for the potential functions of the signatures in the responses to immunotherapy and chemotherapy.

### Model Specificity Analysis of the Prognostic Risk Model

We used the constructed prognostic model for wild-type *LKB1* LUAD. The same analysis used in *LKB1-*mutant LUAD was performed in wild-type *LKB1* LUAD to examine if the prognostic risk model was only applicable to *LKB1-*mutant cancers.

All statistical and modeling analyses were performed using Perl, GSEA (GSEA_4.1.0), and R (version 4.0.2) software.

## Results

**FIGURE 1:**
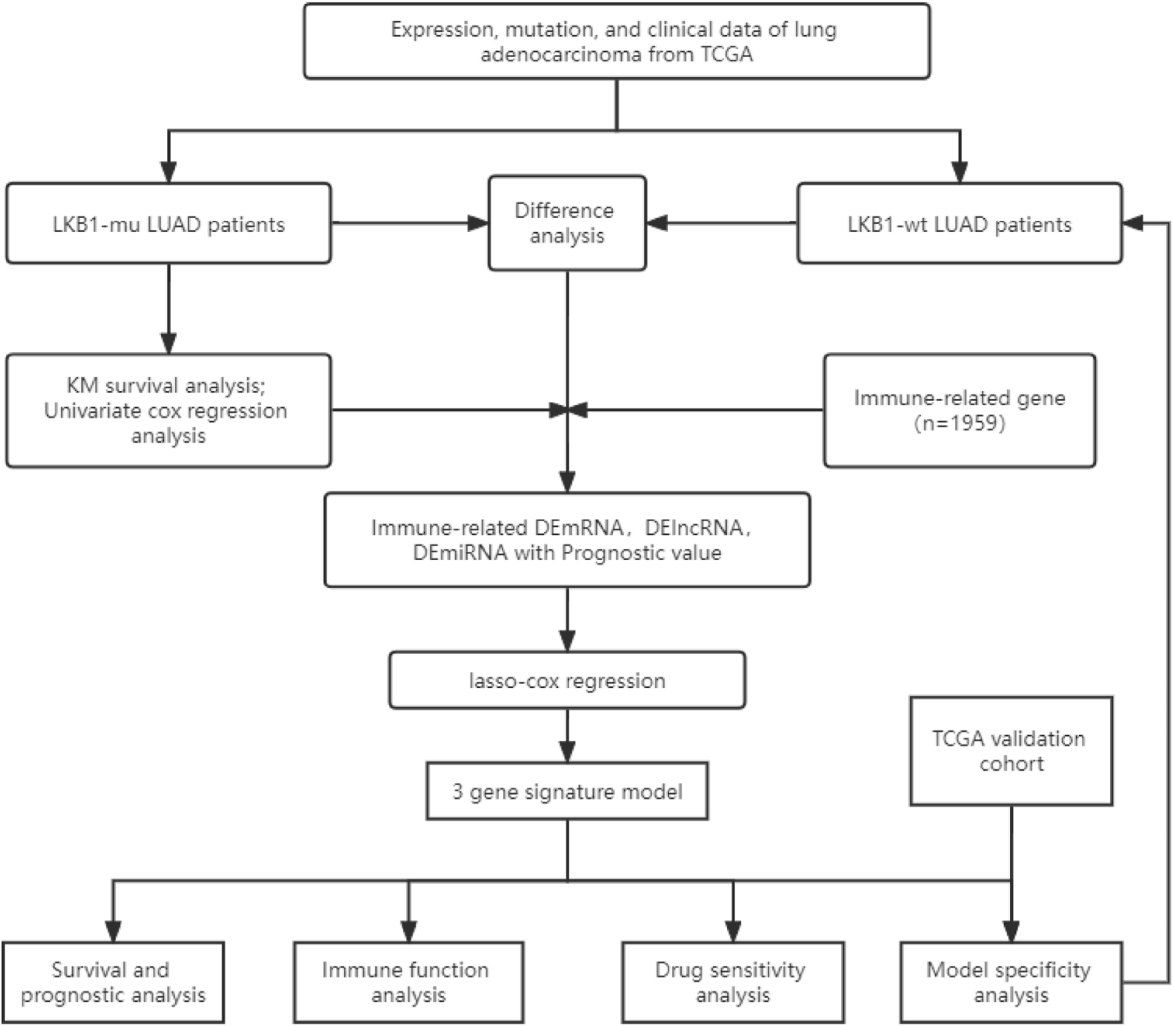
Flow chart of this study.

The flow chart of this study is shown in Figure 1.

### *LKB1* Mutations Suppress the Immune Landscape of Lung Adenocarcinoma

**FIGURE 2:**
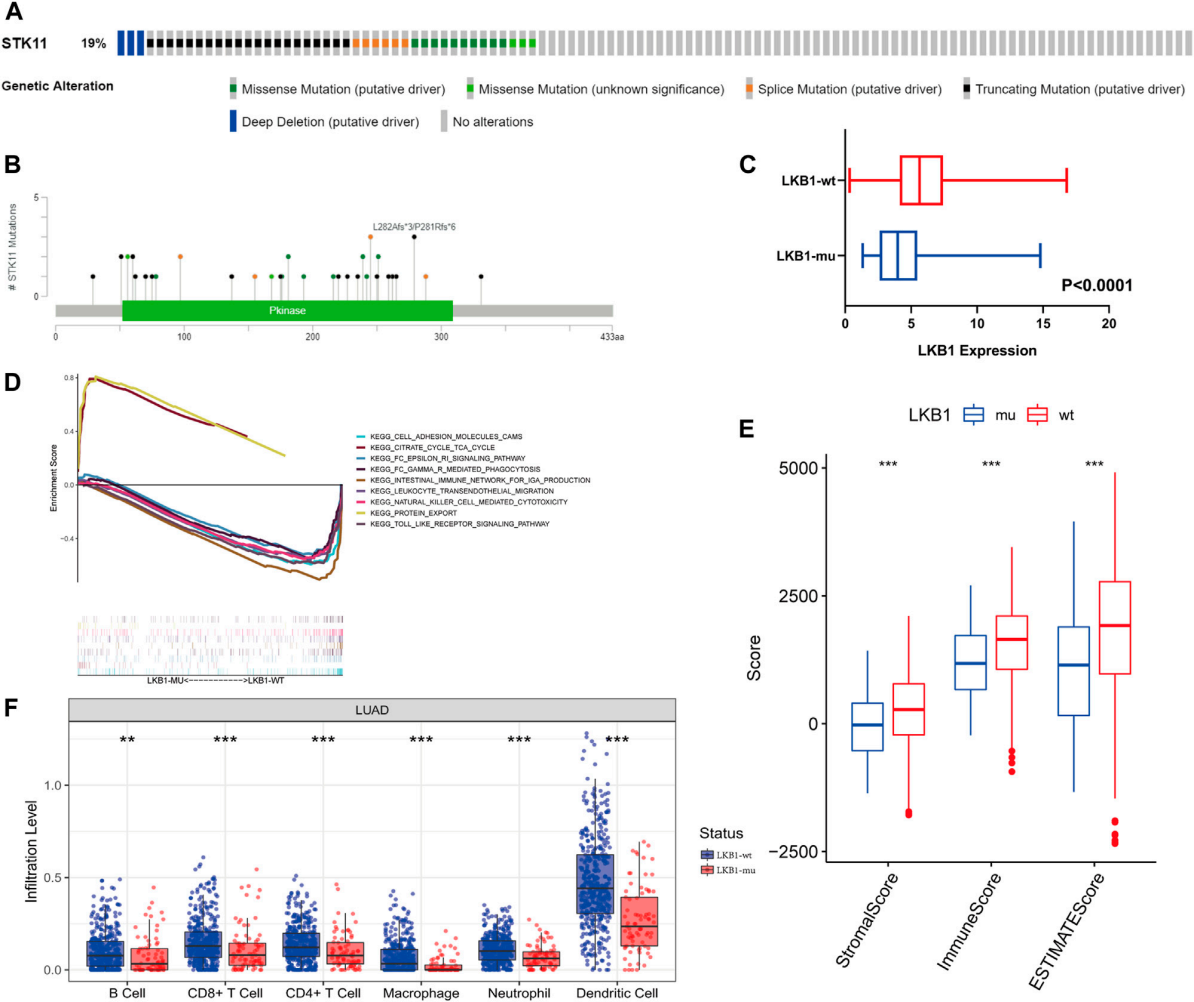
From the cBioPortal database, **(A)** *LKB1* genetic alterations **(B)** and mutation positions in LUAD patients of TCGA database. **(C)** Difference in the expression level of *LKB1* between the LKB1-wt and LKB1-mu groups. **(D)** GSEA in *LKB1*-mutant LUAD. **(E)** ESTIMATE score, immune score, and stromal score and **(F)** infiltration abundance of six kinds of immune cells in the LKB1-wt and LKB1-mu groups.

In TCGA cohort, 19% of LUAD patients were found to possess an *LKB1* mutation (Figure 2A). Missense mutations and truncating mutations were the main mutation types, and these mutations were mostly located in the kinase domain (Figures 2A,B). These mutations significantly affected the expression level and function of LKB1 in cells. These findings were consistent with our hypothesis that the expression level of LKB1 is low in *LKB1-*mutant LUAD (Figure 2C).

Cell adhesion, natural killer cell-mediated cytotoxicity, toll-like receptor signaling pathways, and other immune-related pathways were suppressed in LKB1-mu LUAD as compared with LKB1-wt LUAD (Figure 2D). A lower stromal score, immune score, and ESTIMATE score (all *p* < 0.001) in the LKB1-mu group also suggested a lower degree of immune cell infiltration (Figure 2E, Supplementary Figure S1A). Because different immune cells play different roles in tumor progression, we further explored the differential degrees of infiltration of different types of immune cells. The TME of the LKB1-mu group possessed fewer B cells, CD8^+^ T cells, CD4^+^ T cells, macrophages, neutrophils, and dendritic cells (Figure 2F). Additional details regarding immune cell infiltration are given in Supplementary Figure S1B.

Of the 46 known immune checkpoint genes, 17 were found to be significantly differentially expressed between the two groups of LUAD. These differentially expressed genes included *CD274*, which codes for the programmed death ligand 1 (PD-L1), *CD40*, and *CD44* (Supplementary Figure S1C). Notably, the high expression of PD-L1 LKB1-mutant LUAD is consistent with the results that demonstrate low infiltration of T cells. These results showed that the mutation of *LKB1* was a common event in LUAD and that these mutations suppressed the immune landscape.

### Prognostic Risk Model of a Three-Gene Signature

**FIGURE 3:**
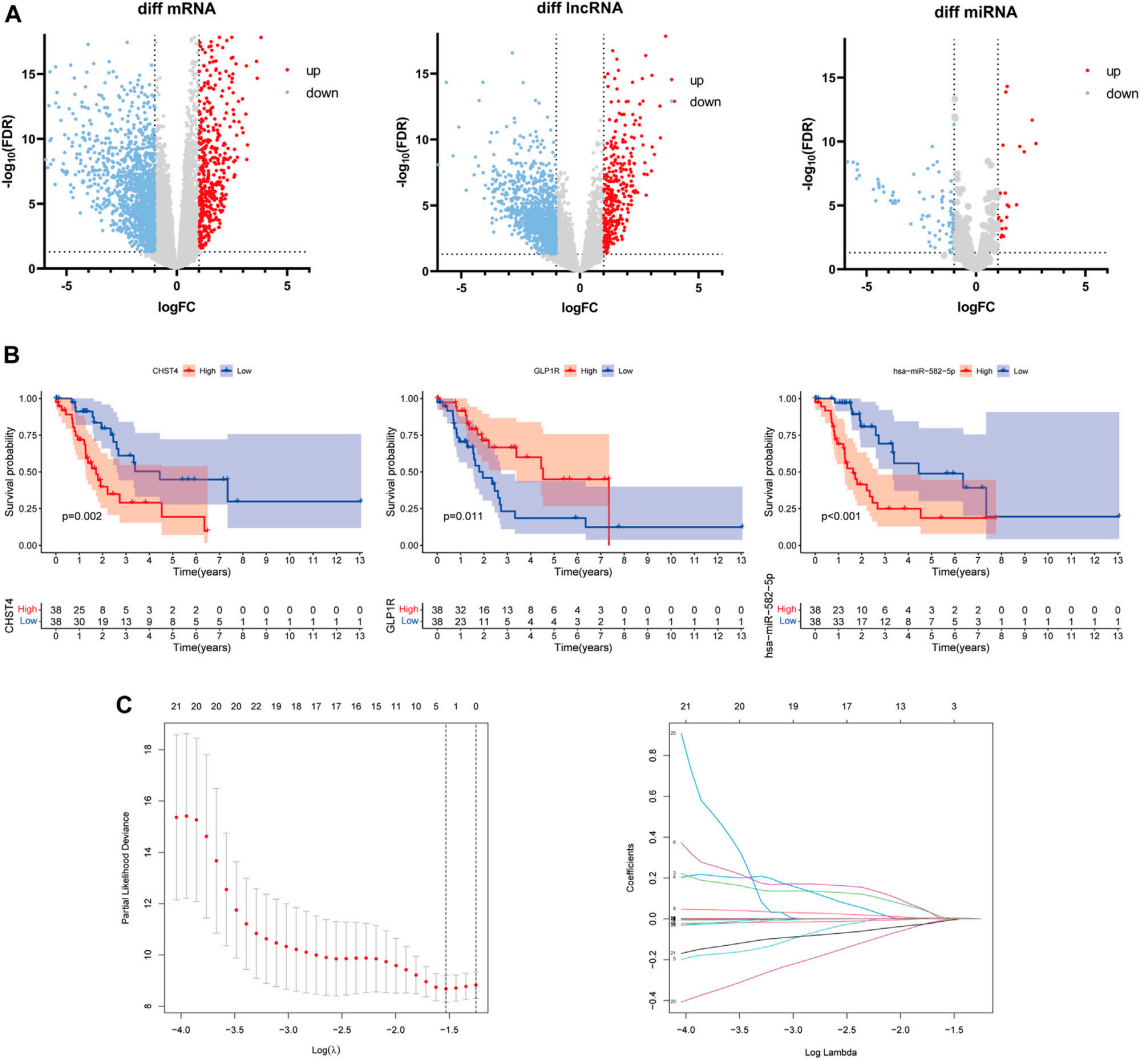
**(A)** Differentially expressed mRNAs, lncRNAs, and miRNAs between the LKB1-wt and LKB1-mu groups. **(B)** Kaplan–Meier survival analysis of candidate signatures. **(C)** Result plots of the LASSO regression analysis.

The differential analysis results uncovered a total of 1,869 DEmRNAs, 1,549 DElncRNAs, and 105 DEmiRNAs (Figure 3A). According to the correlation analysis, the differential expressions of 228 immune-related mRNAs were found to intersect with the differential expressions of 488 immune-related lncRNAs. The K–M analysis and univariate Cox regression analysis further identified a total of 26 mRNAs, 9 lncRNAs, and 8 miRNAs (*p* < 0.05, Figure 3B, Supplementary Tables S1, S2, and S3), for a total of 43 RNAs.

The LASSO-Cox regression analysis was applied to establish a prognostic model using the expression profiles of these 43 differentially expressed genes. A signature including three genes (*CHST4*, *GLP1R*, and hsa-miR-582-5p) was identified based on the optimal value of *λ* (Figure 3C). The risk score was calculated as follows: risk score = 0.0113 × C_e_—0.0817 × G_e_ + 0.0016 × H_e_, where C_e_ is the expression level of *CHST4,* G_e_ is the expression level of *GLP1R,* and H_e_ is the expression level of hsa-miR-582-5p. The *LKB1-*mutant patients were stratified into a high-risk group (*n* = 37) and a low-risk group (*n* = 37) according to the median cut-off value of the risk score.

### Survival and Prognostic Analysis Using the Risk Model

**FIGURE 4:**
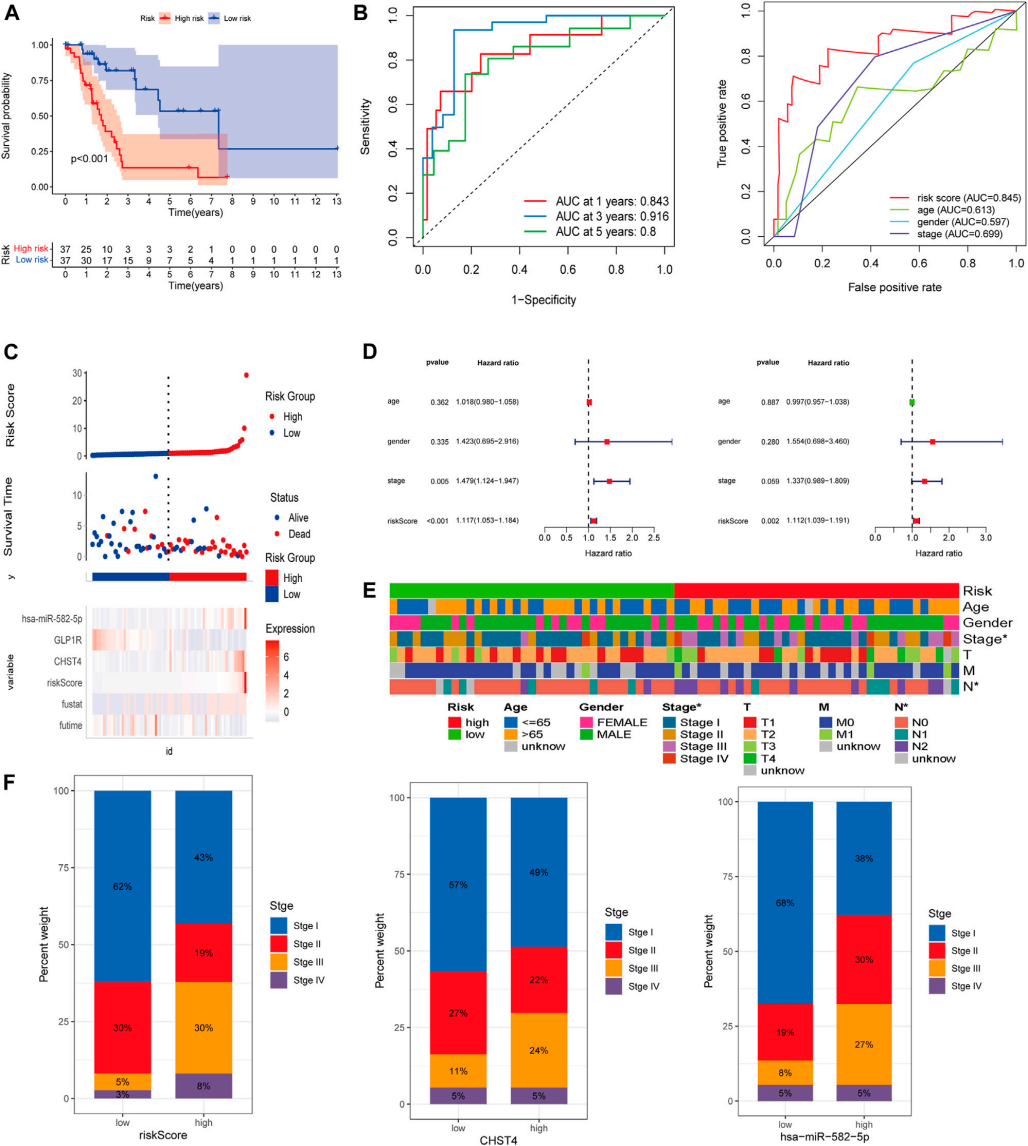
**(A)** Kaplan–Meier survival analysis in the high-risk and low-risk groups. **(B)** ROC curves of the risk score and clinical-pathological factors with OS. **(C)** Risk factor analysis and **(D)** independent prognostic analysis of risk scores in *LKB1*-mutant LUAD. **(E)** Heatmap and **(F)** proportion diagram of clinical correlation analyses.

The K–M curve demonstrated that patients in the high-risk group had a significantly worse OS than patients in the low-risk group (Figure 4A, *p* < 0.001). The 1-, 3-, and 5-year AUC of the risk score for OS were 0.843, 0.916, and 0.8, respectively (Figure 4B). Moreover, the predictive performance of the risk score (AUC = 0.845) was better than the performance of age, gender, and stage (AUC = 0.613, 0.597, and 0.699, respectively) (Figure 4B). As shown in Figure 4C, patients in the high-risk group had a higher probability of death and shorter survival time.

Univariate and multivariate Cox regression analyses also indicated that the risk score could be used as an independent prognostic factor for *LKB1-*mutant LUAD (Figure 4D). The risk score was found to be significantly positively associated with the stage (Figures 4E,F). Figure 4F indicates that higher expression levels of *CHST4* and hsa-miR-582-5p had higher stage patients. In addition, the expression level of hsa-miR-582-5p was found to be associated with T and N (*p* < 0.05, Supplementary Figure S1D).

**FIGURE 5:**
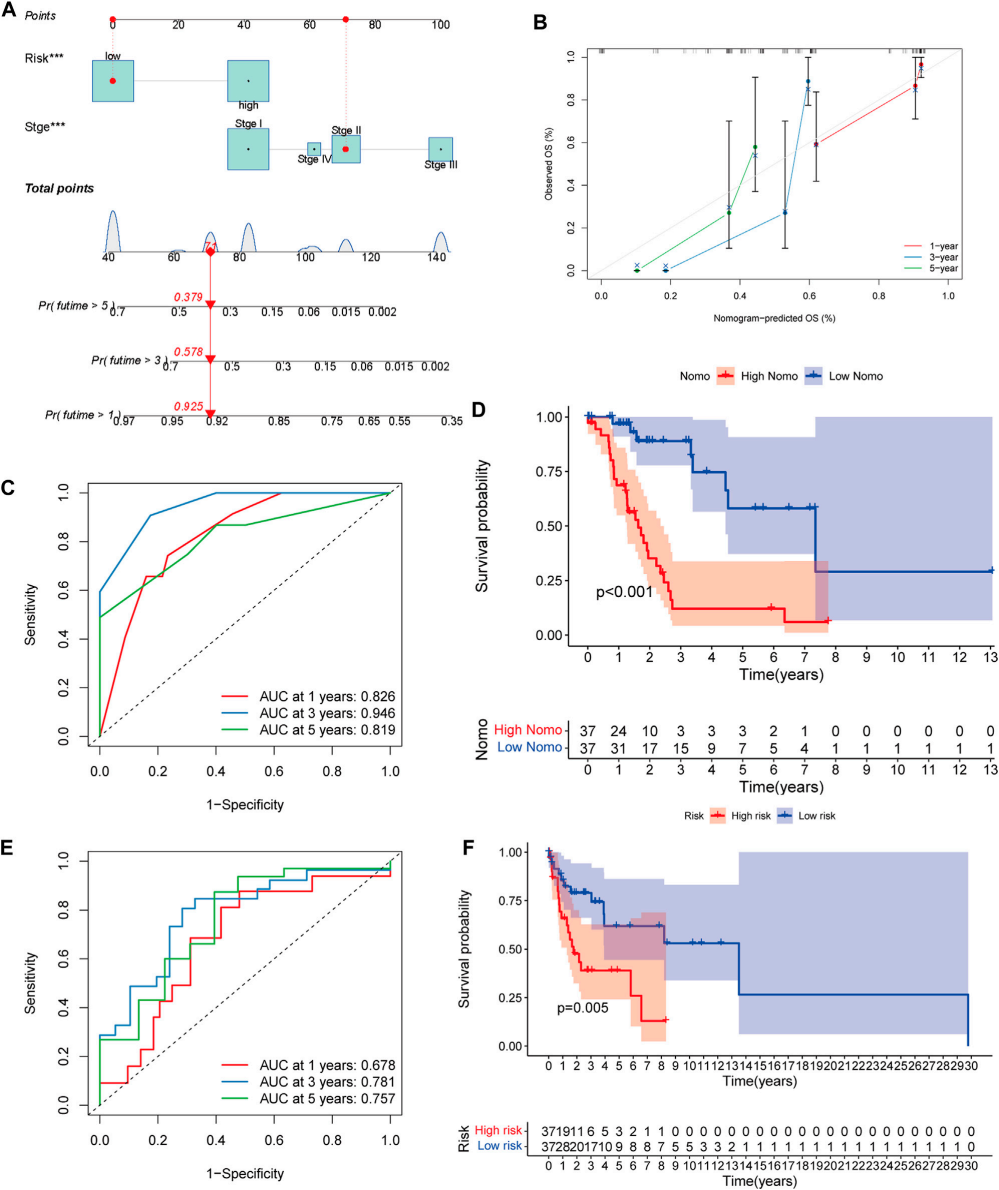
**(A)** Nomogram of *LKB1*-mutant LUAD. **(B)** 1-, 3-, and 5-year calibration plots, **(C)**, 1-, 3-, and 5-year ROC curves, and **(D)**, Kaplan–Meier survival analysis of the nomogram. **(E)**, 1-, 3-, and 5-year ROC curves, and **(F)**, Kaplan‐Meier survival analysis of the validation cohort.

To help clinicians better predict the prognosis of LUAD patients with mutated *LKB1*, a nomogram was constructed using the risk score plus stage (Figure 5A). The prognostic capability of the nomogram was further evaluated by ROC curves, K–M survival analyses, and calibration plots. The degree of fit of the calibration curves corresponding to 1-, 3-, and 5-year survival shows good consistency (Figure 5B). The AUC values of ROC curves corresponding to 1-, 3-, and 5-year survival in *LKB1-*mutant LUAD were 0.826, 0.946, and 0.819, respectively (Figure 5C). The K–M curve demonstrated that patients in the high-risk group had a significantly worse OS than patients in the low-risk group (Figure 4D, *p* < 0.001). These results demonstrated that the risk score and nomogram had a good prognostic ability.

When the validation cohort was divided into high- and low-risk groups according to the constructed model, there was a significant difference in the overall survival (*p* = 0.005, Figure 5F). The 1-, 3-, and 5-year AUC were 0.678, 0.781, and 0.757, respectively (Figure 5E). These indicated that this prognostic model had a good survival prediction ability for *LKB1*-mutated cancers.

### Immune Function Analysis of the Prognostic Risk Model

**FIGURE 6:**
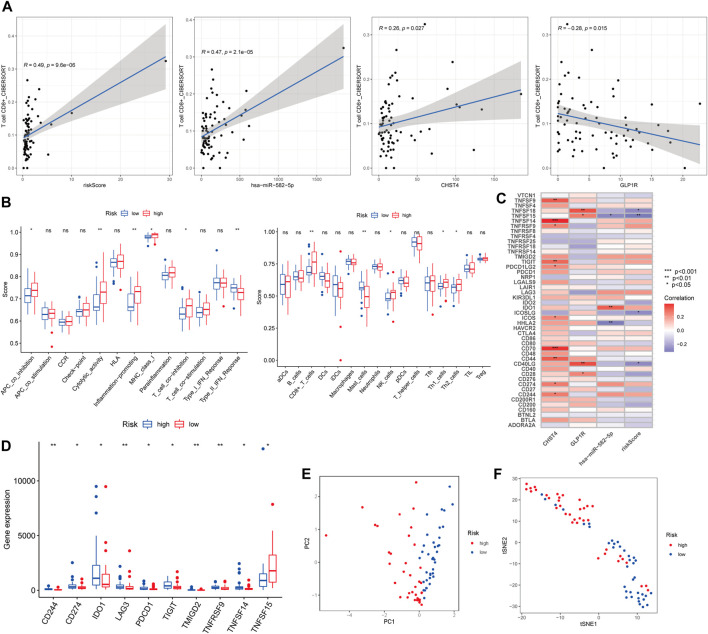
**(A)** Pearson correlation analysis between CD8^+^ T cell and the risk score and three other signatures. **(B)** Boxplots of ssGSEA. **(C)** Correlation analysis and **(D)** boxplots of immune checkpoints. **(E)** PCA and **(F)** t-SNE analysis of risk scores.

The Pearson correlation analysis indicated the risk score and the three other genes were significantly related to CD8^+^ T cell infiltration (R = 0.49, 0.47, 0.26, and −0.28, respectively). Among these factors, *GLP1R* showed a significant negative correlation (*p* < 0.05, Figure 6A). The risk score was associated with four immune cells and five immune-related pathways according to the ssGSEA (Figure 6B). *CHST4*, *GLP1R*, hsa-miR-582-5p, and risk scores were significantly associated with multiple immune checkpoints (Figure 6C). Of the 46 immune checkpoints, 10 were significantly differentially expressed between the high- and low-risk groups of patients with *LKB1-*mutant LUAD (Figure 6D and Supplementary Figure 1E). the PCA and t-SNE analysis demonstrated that the patients were well distinguished according to the risk score (Figures 6E,F).

### Drug Sensitivity Analysis of the Prognostic Risk Model

**FIGURE 7:**
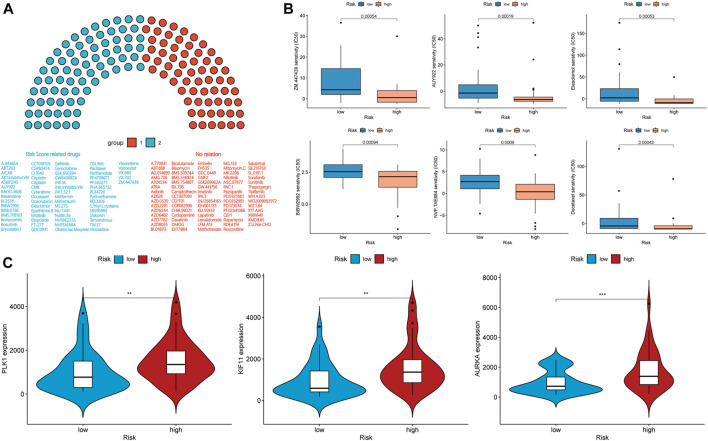
**(A,B)** Drug sensitivity analysis and **(C)** expression level correlation analysis in the high-risk and low-risk groups.

We calculated the half-maximal inhibitory concentration (IC_50_) of 138 chemotherapy drugs in the high- and low-risk groups. The associations of the values of IC_50_ of 64 chemotherapy drugs with the groups were statistically significant (*p* < 0.05), and the *p* values associated with 15 chemotherapy drugs were less than 0.001 (Figures 7A,B and Supplementary Figure S2A). For these chemotherapy drugs, we analyzed the relationship between the risk score and the expression of genes coding for immune checkpoint-related drug targets. The expressions of *AURKA*, *CD274*, *KIF11,* and *PLK1*, which encode targets of ZM.447,439, PD-L1 immunotherapy, S-trityl-l-cysteine, and BI.2536, respectively, were associated with the risk score (Figure 7A, Figure 6D). However, the expression level and mutation status of *EGFR* were not associated with the risk score (Supplementary Figure S2B). The risk score was not statistically correlated with the targets of other drugs and HLA-related antigens (Supplementary Figure S2B).

### Model Specificity Analysis of the Prognostic Risk Model

**FIGURE 8:**
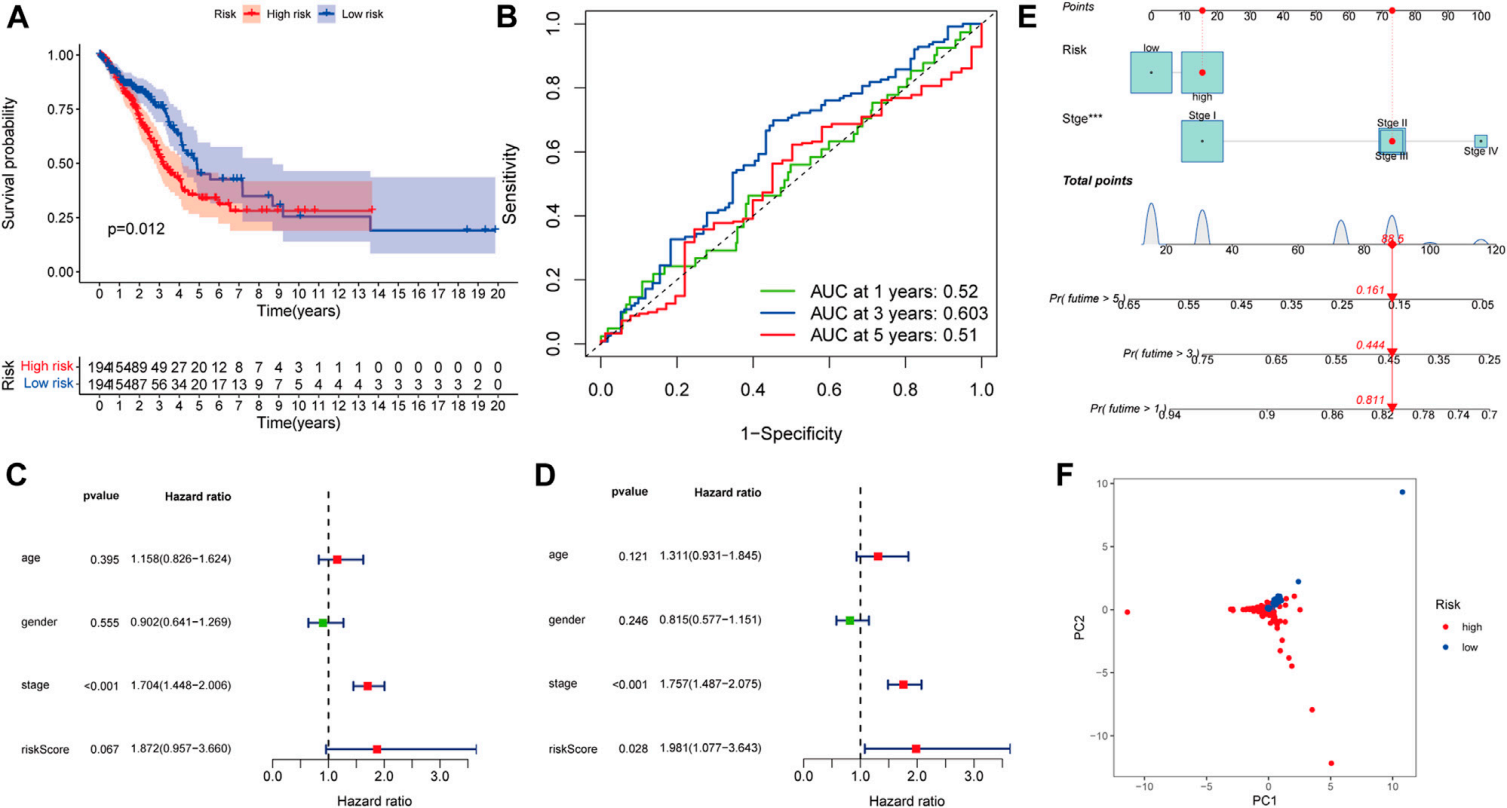
Model specificity analysis of the prognostic risk model: **(A)** Kaplan–Meier survival analysis , **(B)** ROC curves , **(C)** univariate and **(D)** multivariate Cox regression analyses. **(E)** Nomogram of LKB1-wild-type LUAD. **(F)** PCA.

When we applied the same risk model to the *LKB1*-wt group, the K–M curve demonstrated that patients in the high-risk group had a worse OS than patients in the low-risk group (Figure 8A, *p* = 0.012). However, the 1-, 3-, and 5-year AUC of the risk score for the OS were 0.52, 0.603, and 0.51, respectively (Figure 8B), which showed that the predictive performance of the risk score was poor. According to univariate and multivariate Cox regression analyses, the risk score could not be used as an independent prognostic factor for LUAD patients with wild-type *LKB1* (Figures 8C,D). LUAD patients with wild-type *LKB1* could not be distinguished according to the risk score (Figure 8F). There was no obvious relationship between the risk score and probability of death (Supplementary Figure S3A).

The same nomogram was also constructed using the risk score plus stage in *LKB1-*wild-type patients (Figure 8E). However, the impact of the risk score on patient prognosis was much smaller than the impact of the stage. The calibration curves corresponding to 1-, 3-, and 5-year survival rates show consistency (Supplementary Figure S3B). The AUC values of ROC curves corresponding to 1-, 3-, and 5-year survival in LKB1-wt UAD were 0.701, 0.718, and 0.691, respectively (Supplementary Figure S3C). The K–M curve demonstrated that patients in the high-risk group had a significantly worse OS than patients in the low-risk group (Supplementary Figure S3D, *p* < 0.001). But depending on the nomogram, the stage plays a more significant role than the risk score, resulting in the nomogram showing better predictive ability. These results demonstrated that the risk score was not applicable to wild-type *LKB1* tumors.

## Discussion

LUAD patients diagnosed at the same stage according to the current TNM classification system can have different clinical outcomes, due to heterogeneity at both the molecular and the genetic levels (Navin et al., 2011; Gerlinger et al., 2012). Notably, *LKB1-*inactivating mutations are important cancer drivers and have a significant impact on the prognosis (Tsai et al., 2014). Because of the extensive tumor suppressor function and unique effect on the immunity of *LKB1*, the treatment of *LKB1*-mutant LUAD is progressing slowly. We therefore conducted this research to explore the immune landscape, construct an immune-related prognostic risk model, and predict effective drugs for the treatment of *LKB1*-mutant LUAD.

As a major driver of the cold non-T cell-inflamed microenvironment, inactivating *LKB1* genomic alterations cause a decrease in the infiltration of CD3^+^, CD4^+^, and CD8^+^ T cells and low expression levels of PD-L1 (Skoulidis et al., 2015; Scheel et al., 2016; Kadara et al., 2017; Skoulidis et al., 2018). These findings are consistent with our research. Mechanistically, loss of *LKB1* correlates with increases in the expression of IL-1β, IL-6, CXCL7, and G-CSF that promote myeloid cell recruitment and induce epigenetic repression of STING (Koyama et al., 2016; Kitajima et al., 2019). Corresponding increases in the expression of T-cell exhaustion markers and tumor-promoting cytokines result in the inhibition of T cells under the condition of *LKB1* deficiency (Koyama et al., 2016). Thus, loss of *LKB1* is not only related to the resistance to PD-1 and PD-L1 inhibition, but also to the resistance to combined anti-PD-1 and anti-CTLA4 therapy with nivolumab and ipilimumab (Hellmann et al., 2018). According to a research work by Koyama et al. (2016), treatment with an IL-6-neutralizing antibody or a neutrophil-depleting antibody yielded therapeutic benefits in *LKB1*-deficient patients. These results indicate that *LKB1* has an important impact on immune function, and further exploration is necessary.

Therefore, we endeavored to identify specific RNAs associated with immunity and the survival prognosis. In this study, we identified a three-gene prognostic model associated with immunity that was constructed using TCGA datasets to predict the OS outcomes of patients with *LKB1*-mutant LUAD. These same three genes have been identified as prognostic biomarkers in multiple tumor types, and have been found to play important roles in the occurrence and development of various diseases by regulating immune function (Moschovaki Filippidou et al., 2020; Zhang et al., 2020; Hu et al., 2021; Ren et al., 2022). The AUC values of ROC curves suggested that our risk model had a superior prognostic value to other clinicopathological characteristics, including age, gender, and stage. According to univariate and multivariate Cox regression analyses, the prognostic risk model that included the three immune-related RNAs was the only independent prognostic factor for patients with *LKB1*-mutant LUAD.

Two of the three immune-related RNAs and the risk score had significant associations with the stage. More interestingly, the expression of hsa-miR-582-5p was associated with tumor size and lymph node status, implying that the risk score is not only strongly linked to the intrinsic biological characteristics of *LKB1*-mutant LUAD, but also that it has a close relationship with clinicopathological characteristics. The same analysis in LKB1-wt lung adenocarcinoma showed a poor survival predictive power. It is worth noting that the importance of the risk score and stage in influencing the prognosis was opposite in nomograms created for *LKB1*-mutated and wild-type LUAD.

In addition to excellent prognostic prediction ability, the risk score is closely related to immune function. The upregulation of natural killer cells in the high-risk group might be because of the dual role of these cells regarding both cancer progression and the boosting of the onset of immuno-suppressant TMEs (Di Vito et al., 2019). Increased levels of CD8^+^ cells have been shown previously to be associated with better outcomes in non-small-cell lung carcinoma, and CD8^+^ cells were an independent prognostic factor in this analysis (Fehniger et al., 2003). However, in our analysis, more CD8^+^ cells and higher cytolytic activity were found in the high-risk group. More detailed research thus needs to be performed to explore the underlying mechanisms. Moreover, higher risk scores correlated with protumor immunity, including the activity of inflammation-promoting and APC co-inhibition, and these factors may provide an explanation for their poor prognosis.

We used a drug sensitivity analysis to compare the IC_50_ values of the high-risk and low-risk groups to confirm the ability of the risk score as a treatment-guiding biomarker. Among the three EGFR tyrosine kinase-inhibitors investigated, gefitinib, erlotinib, and afatinib (BIBW2992), only afatinib was found to have a statistically significant difference in efficacy between the high- and low-risk groups. However, the expression level and mutation status of EGFR were not related to the risk score. We speculated that this disparate result may be caused by the additional activity of afatinib against Her2, which has been investigated in the context of breast cancer and other EGFR and Her2-driven cancers (Lin et al., 2012). According to the value of IC_50_ and the correlation between drug targets and risk scores, ZM.447,439, an aurora kinase-inhibitor, is the most appropriate treatment for patients in the high-risk group. Meanwhile, we noticed that the IC_50_ of the high-risk group was often lower than that of the low-risk group, and the expression level of PD-L1 is also higher in the high-risk group; this correlation means that patients in the high-risk group are more sensitive to some drugs despite having more malignant characteristics. This conclusion demonstrates the importance of identifying high-risk patients through risk scores to identify effective treatments and to investigate prognoses of LUAD patients with *LKB1* mutations.

However, there are several limitations to our study. First, because of the lack of data on patients with *LKB1*-mutant LUAD, the sample size of the present study is not as large as other studies, which may influence the precision of the results. Second, the underlying molecular mechanisms that would explain why the risk score can predict the prognosis of patients with *LKB1*-mutant LUAD have not been fully clarified. It is necessary for us to explore the links between the risk score and immune activity by conducting experiments. In addition, large-scale and multi-center studies are needed to confirm our results.

## Conclusion

The risk model we constructed provides a new promising biomarker that can provide precise clinical insights into the progression of LUAD in patients with *LKB1* mutations and can serve as an efficient guide for the treatment of this challenging disease.

## Data Availability

Publicly available datasets were analyzed in this study. These data can be found at: https://www.cancer.gov/about-nci/organization/ccg/research/structural-genomics/tcga.

## Abbreviations

AUC, area under the curve; FDR, False discovery rate; GSEA, gene set enrichment analysis; HR, hazard ratio; IC50, half maximal inhibitory concentration; ICI, immune checkpoint; LncRNA, long non-coding RNA; LUAD, lung adenocarcinoma; miRNA, microRNA; OS, overall survival; PCA, principal component analysis; ROC, receiver operating characteristic; ssGSEA, single-sample gene set enrichment analysis; TCGA, The Cancer Genome Atlas; TME, tumor microenvironment; t-SNE, t-distributed stochastic neighbor embedding.
