# Supplementary material for: The Prognostic Model and Drug Sensitivity of LKB1-Mutant Lung Adenocarcinoma Based on Immune Landscape
Source: Front Mol Biosci. 2022 Jun 2;9:756772. doi: 10.3389/fmolb.2022.756772 (PMC9201220; doi:10.3389/fmolb.2022.756772)
Supplement: Supplementary file 1 [file Table1.DOCX]

Supplementary Material

# 1 Supplementary Figures and Tables

**1.1 Supplementary Figures**

**
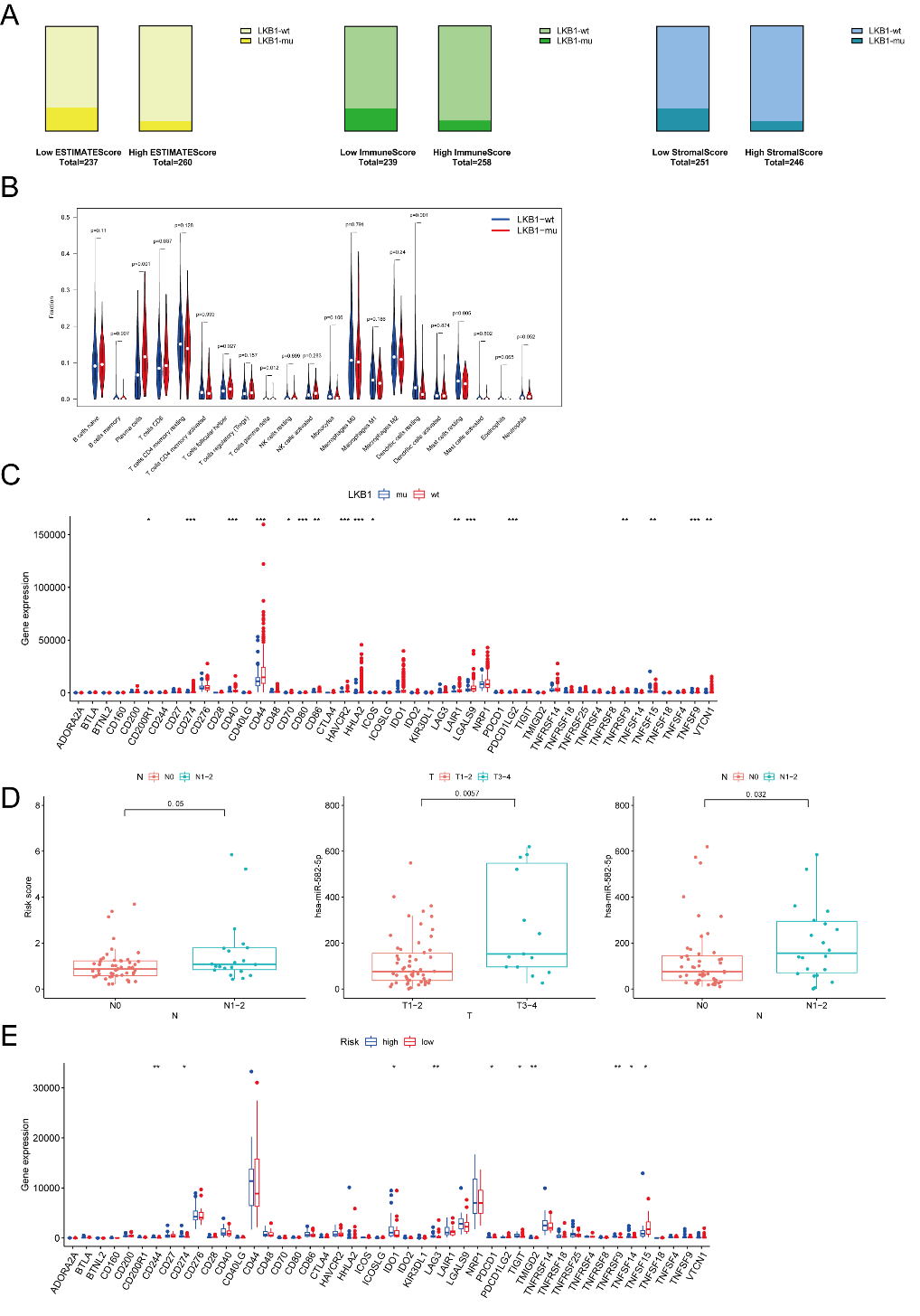
**

**Supplementary Figure 1.** A. ESTIMATE Score, Immune Score, Stromal Score ratio graph. B. Violin plot of the infiltration abundance of 21 kinds of immune cells in LKB1-wt and LKB1-mu groups. C. Different expression of immune checkpoint between LKB1-mu and LKB1-wt LUAD. D. boxplots of clinical correlation analysis. E. Different expression of immune checkpoint between high- and low-risk LUAD.


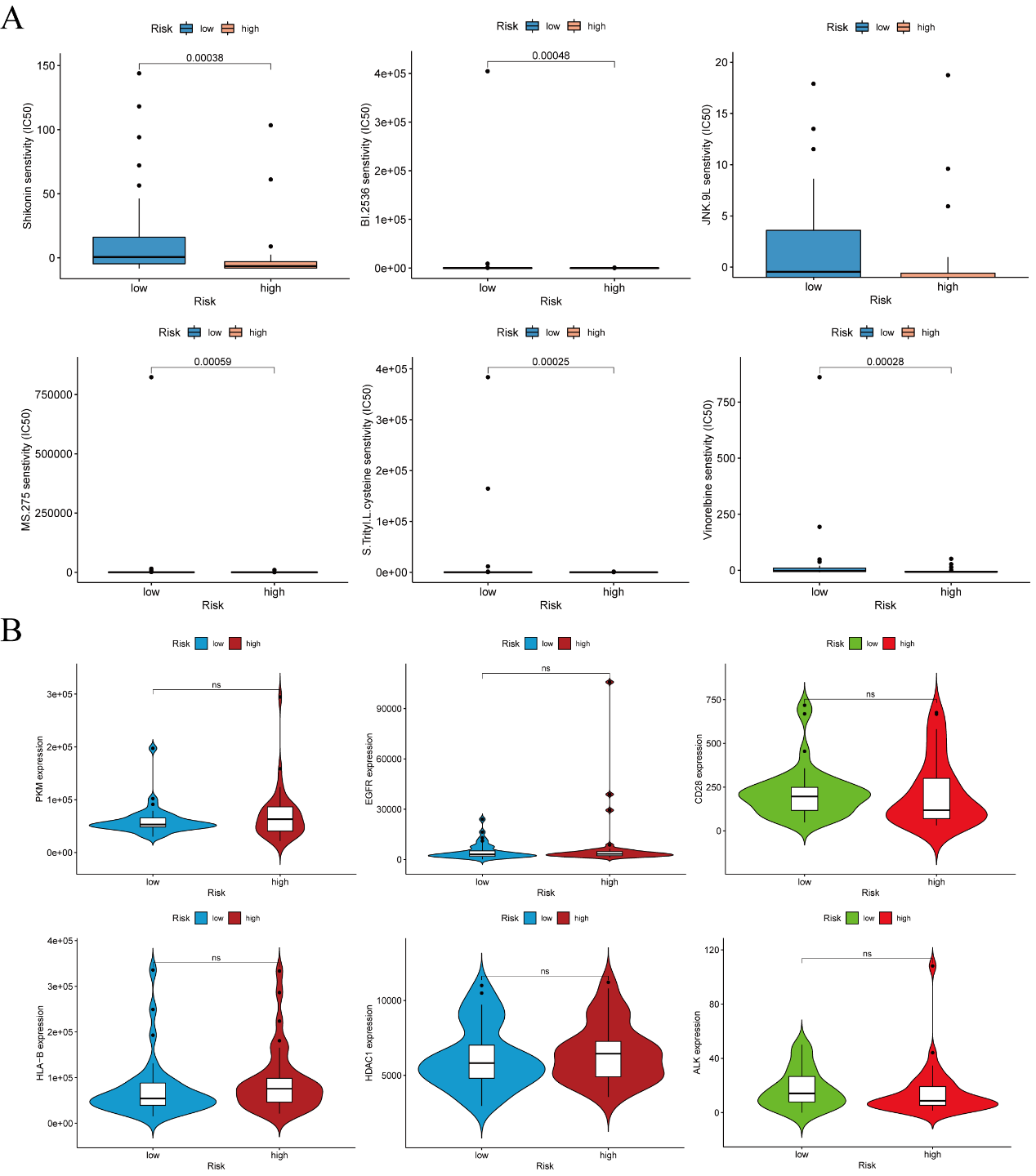


**Supplementary Figure 2.** Drug sensitivity analysis (A) and expression level correlation analysis (B) in the high-risk and low-risk groups.


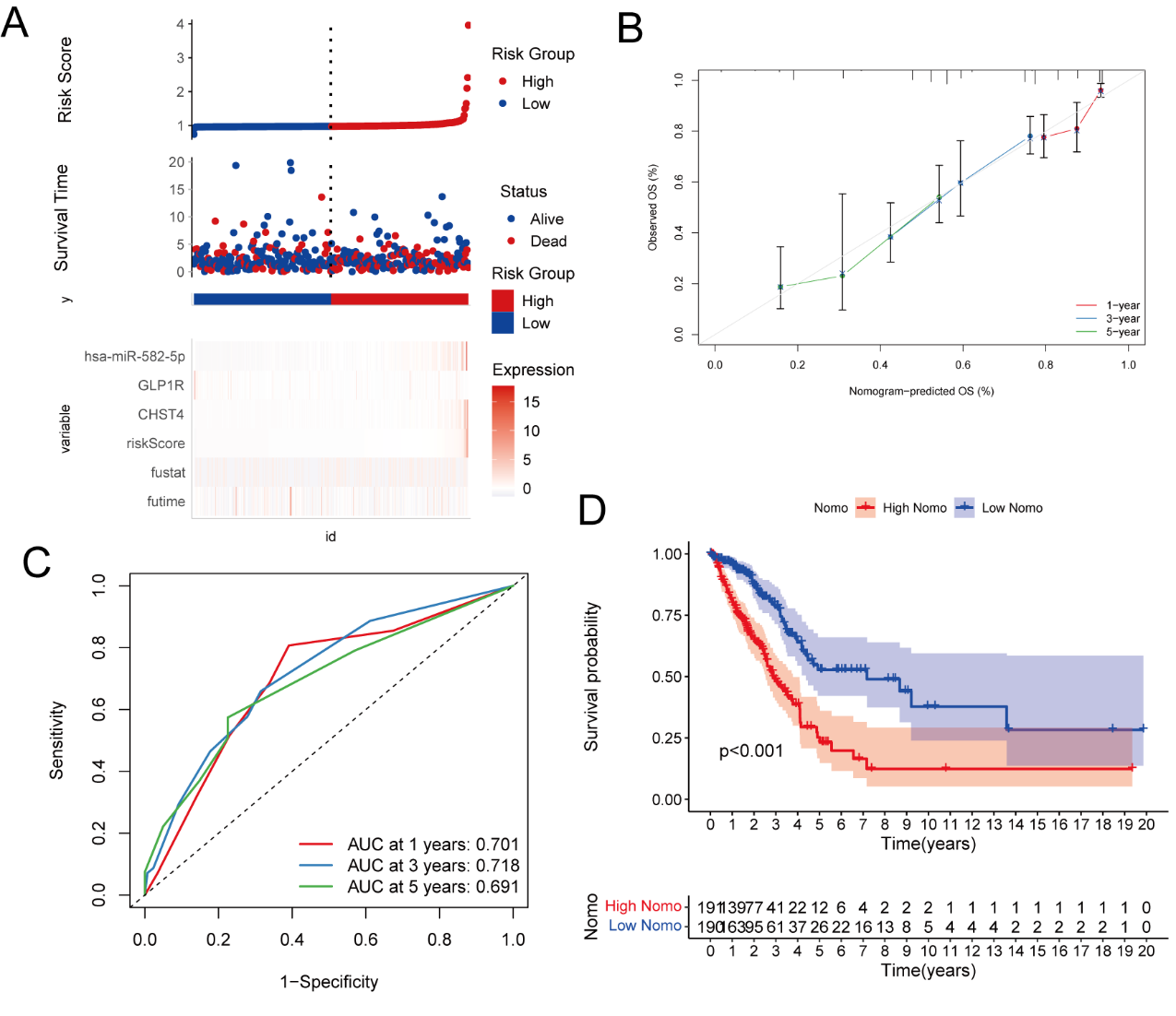


**Supplementary Figure 3.** Risk factor analysis (A), 1-, 3-, and 5-year calibration plots (B), ROC curves (C), KM survival analysis (D) of the nomogram of LKB1 wild LUAD.

**1.2 Supplementary Tables**

Supplementary Table 1: Immune-related DEmRNA with prognostic value

| mRNA | KM survival analysis | Univariate cox regression analysis | | |
| --- | --- | --- | --- | --- |
|  | P value | HR | | P value |
| APOA1 | P<0.001 | 0.661 | 0.001 | |
| AREG | 0.009 | 1.381 | 0.002 | |
| BMP3 | 0.040 | 0.769 | 0.005 | |
| BPIFB2 | 0.029 | 0.876 | 0.002 | |
| CCL17 | 0.011 | 0.734 | 0.009 | |
| CHST4 | 0.002 | 1.641 | P<0.001 | |
| CRLF1 | 0.032 | 0.848 | 0.001 | |
| CRP | 0.010 | 1.357 | 0.016 | |
| CX3CL1 | 0.001 | 0.536 | 0.001 | |
| DPP4 | 0.039 | 0.742 | 0.002 | |
| EDN3 | 0.031 | 0.804 | 0.031 | |
| EPPIN | 0.038 | 0.759 | 0.014 | |
| EREG | P<0.001 | 1.319 | P<0.001 | |
| FGF5 | 0.043 | 1.467 | P<0.001 | |
| GLP1R | 0.011 | 0.730 | 0.009 | |
| HRG | 0.007 | 1.276 | 0.013 | |
| KL | 0.033 | 0.620 | 0.001 | |
| KLRC2 | 0.008 | 1.434 | P<0.001 | |
| LGR5 | 0.008 | 0.698 | 0.001 | |
| MSTN | 0.008 | 0.768 | 0.043 | |
| PCSK2 | 0.004 | 0.905 | 0.004 | |
| PGC | 0.001 | 0.905 | 0.001 | |
| PROC | 0.010 | 0.817 | 0.015 | |
| PROK1 | 0.035 | 0.564 | 0.004 | |
| SCGB3A1 | 0.004 | 0.891 | 0.030 | |
| TENM1 | 0.021 | 0.904 | 0.041 | |

Supplementary Table 2: Immune-related DElncRNA with prognostic value

| LncRNA | KM survival analysis | Univariate cox regression analysis | | |
| --- | --- | --- | --- | --- |
|  | P value | HR | | P value |
| AC002558.3 | 0.025 | 1.874 | 0.010 | |
| AC010547.2 | 0.032 | 1.747 | 0.000 | |
| AC022973.3 | 0.006 | 0.560 | 0.004 | |
| AC103876.1 | 0.035 | 0.503 | 0.046 | |
| AL136320.1 | 0.004 | 1.826 | 0.004 | |
| BTBD9-AS1 | 0.037 | 0.558 | 0.006 | |
| LINC00707 | 0.019 | 1.206 | 0.002 | |
| LINC01133 | 0.032 | 1.213 | 0.005 | |
| WT1-AS | 0.010 | 1.266 | 0.011 | |

Supplementary Table 3: Immune-related DEmiRNA with prognostic value

| miRNA | KM survival analysis | Univariate cox regression analysis | |
| --- | --- | --- | --- |
|  | P value | HR | P value |
| hsa-miR-1251-5p | P<0.001 | 0.733 | 0.004 |
| hsa-miR-135a-5p | 0.014 | 0.807 | 0.034 |
| hsa-miR-196b-5p | 0.012 | 1.232 | 0.005 |
| hsa-miR-34b-5p | 0.027 | 0.737 | 0.005 |
| hsa-miR-431-3p | 0.047 | 1.305 | 0.039 |
| hsa-miR-520f-3p | 0.018 | 4.357 | 0.004 |
| hsa-miR-582-3p | P<0.001 | 1.544 | P<0.001 |
| hsa-miR-582-5p | 0.001 | 1.502 | P<0.001 |
